# Rapid visual detection of *FecB* gene expression in sheep

**DOI:** 10.1515/biol-2020-0091

**Published:** 2020-12-24

**Authors:** Li Liu, Ruirui Hu, Cunyuan Li, Xiaoyue Li, Wei Ni, Rui Yao, Mengdan Zhang, Huixiang Li, Yueren Xu, Yaseen Ullah, Shengwei Hu

**Affiliations:** College of Life Sciences, Shihezi University, Shihezi, Xinjiang, 832003, China; College of Animal Science and Technology, Shihezi University, Shihezi, Xinjiang, 832003, China

**Keywords:** ARMS, *FecB* gene, SYBR Green I, mismatch

## Abstract

Sheep play an important role in agricultural production and people’s lives, and fecundity is one of the most important economic traits of sheep for sheep breeders. The Booroola fecundity (*FecB*) gene has a certain correlation with litter size in sheep. Therefore, this study aims to detect *FecB* expression quickly, accurately and visually. Here, we used the nucleic acid dye SYBR Green I to detect *FecB* with the amplification refractory mutation system (ARMS), which can efficiently, rapidly, economically and visually detect *FecB* expression in sheep. After ARMS polymerase chain reaction (PCR), SYBR Green I was directly added to the ARMS products, and whether the sheep carried *FecB* was judged by directly observing the color change in the PCR tube. Homozygous (BB) or heterozygous (B+) samples with *FecB* mutation were bright green, while wild type (++) samples without *FecB* were orange yellow. This study suggested that this method has 100% accuracy and 0.5 ng/µL sensitivity. To our knowledge, this is the first report that shows the integration of the ARMS with SYBR Green I to detect *FecB* expression in sheep visually.

## 1 Introduction

Sheep play an important role in agricultural production and the daily lives of humans. They are very important livestock animals. Fertility and reproductive efficiency are two key factors affecting the economy of the sheep breeding industry [1], and the most important economic trait in sheep farming is fertility. Relevant studies have shown that the major fecundity genes can significantly increase the number of multiple births, thereby improving the reproductive efficiency of sheep and economic position of sheep farmers [2,3]. However, the vast majority of sheep breeds produce single lambs and a few twin lambs. This greatly affects the litter size of ewes, which is the most important reproductive indicator for sheep production. *FecB* (Booroola fecundity) is a major gene, which was originally found in Australian Merino sheep named Booroola during the 1980s, which can increase ovulation and litter size. It was the first major fecundity gene identified in sheep [4,5]. Previous research [6,7,8,9,10] has found that the litter size is positively correlated with the copy number of the *FecB* gene, that is, it is correlated with the genotype. In different sheep breeds and strains, the *FecB* gene distribution is uneven (Table 1). However, the *FecB* gene is associated with high sheep yield. It has been reported [6] that the high fecundity of sheep carrying *FecB* was the result of *FecB* mutation. Additional research [7] has showed that the heterozygote (B+) resulted in 0.93 more lambs than the wild type (++) in sheep [7] and that the B+ genotype also had a 65.6% higher litter size than the ++ genotype [8]. Further studies [9,10] have shown that sheep with the BB genotype or B+ genotype had significantly higher litter size than those with the ++ genotype.

**Table 1:** Frequency distribution of *FecB* in sheep around the world

Breed/strain	Allele frequency		Country	References
	B	＋		
Hu	0.949	0.051	China	[9]
Small Tail Han	0.735	0.265	China	[11]
Chinese Merino prolific strain	0.48	0.52	China	[12]
Nilagiri	0.14	0.86	India	[3]
Kendrapada	0.73	0.27	India	[13]
Kalehkoohi	0.35	0.65	Iran	[14]

Community-based sheep breeding programs (CBBPs) have been reported to generate substantial genetic gains and socioeconomic benefits [15]. It is believed that CBBPs are technically feasible, and it can bring considerable genetic gains in performance features and affect farmers’ livelihoods. Since there is no direct breeding, the number of individuals with *FecB* is relatively small, and most of them are still scattered in the population. If we intentionally strengthen the breeding of the BB genotype or B+ genotype according to the breeding measures of this breed to establish a nucleus herd, we can increase the frequency of the BB genotype or B+ genotype and fix the *FecB* gene. Therefore, rapid and accurate visual detection of *FecB* gene expression in sheep may provide an effective technique for identifying BB or B+ genotype populations.

*FecB* has a single nucleotide polymorphism (SNP) located in the bone morphogenetic protein receptor IB (*BMPR-IB*) gene. Its mutation site is located at position 746 of the coding region with a change in A → G, causing the 249th amino acid to change from glutamine to arginine (Q249R). Q249R is located between the GS domain (a serine-rich and glycine-rich domain) and the L45 loop of *BMPR-IB*, which is a highly conserved intracellular kinase signal region of *BMPR-IB*, demonstrating that 249R is the *FecB* allele, that is, the *FecB* gene is actually the *BMPR-IB* gene [16]. At present, there are many methods for detecting the *FecB* gene. For example, polymerase chain reaction (PCR)-RFLP [17] is widely used in analyzing the mutation of the *FecB* gene, which needs a series of steps such as PCR amplification, enzyme digestion, electrophoresis, etc. Moreover, one primer of the PCR-RFLP carries a restriction enzyme site, which requires a restriction enzyme (*Ava*II) for digestion of the PCR product. Therefore, this method is time-consuming and the detection cost is too high to be further applied. Other methods, such as DNA sequencing or PCR-SSCP, have trade-offs in terms of accuracy, cost, resolution, time consumption and labor. In addition, the SNaPshot assay is easy to implement, but this method is mainly used for SNP typing of medium flux [18]. Furthermore, this method requires the use of a sequencing instrument to analyze the results and is not suitable for on-site detection. The TaqMan assay requires a large amount of sample DNA and exhibits limited selectivity for low-level mutations [19]. Moreover, this method requires expensive fluorescent probes. These disadvantages become unacceptable when a larger sample size needs to be tested. Compared with other molecular diagnosis methods, the amplification refractory mutation system (ARMS) can produce results within 3 h and is simple, rapid, accurate and specific. The ARMS technology is widely used in genetics, forensic medicine and other related fields. It is a unique, highly sensitive and specific method to detect SNP loci in genes and can be carried out in general molecular biology laboratories and clinical laboratories [20,21].

First, based on the principle of amplification refractory mutation, ARMS specific primers were designed for the mutation sites of the target fragment. The last base of the 3′ end of the primer has to match the mutation site of the target fragment. The principle is that DNA fragments in the PCR are obtained according to the principle of complementary base pairing. Sometimes, the PCR can be carried out when the primers differ from the template by a single base; however, when the two juxtaposed bases at the 3′ end are not complementary to the template, the reaction will be hindered by the difficulty in forming the phosphodiester bond. Therefore, in this study, we designed ARMS specific primers for the SNP loci of the *FecB* gene. For ARMS primers, there is only one base difference from the mutant template, while there are two unmatched bases with the wild type template [20]. Based on this evidence, the last base of the 3′ end of the ARMS reverse primer was designed to be complementary to the *FecB* gene mutation base, and an additional mismatch base was introduced into one of the last five nucleotides at the 3′ end of the ARMS reverse primer intentionally. This significantly decreased the binding rate of the primer to the wild type template, thereby increasing the primer pair’s specificity [22]. The addition of extra mismatch, coupled with the presence of a natural mismatch at the 3′ end, resulted in a significant reduction in the production of PCR products from non-specific alleles, but it had a relatively small impact on the amplification of specific alleles. This modification resulted in a significant increase in the specificity of the primers.

In the method for detecting SNP, the SNaPshot cost per sample is $2 per reaction (Table 2), excluding PCR and purification cost [23]. The cost of PCR-RFLP is about $0.52, and the method operation is more cumbersome [24]. Here, we describe a sensitive, accurate and less expensive method for visual detection of *FecB* gene expression, using SYBR Green I [25] as a nucleic acid dye to stain ARMS amplification products. This method allows to efficiently, rapidly, economically and visually detect *FecB* gene expression in sheep and provides the basis for the selection of high-yielding sheep. Moreover, the visual detection method we established costs less than $0.5 per sample.

**Table 2:** Comparison of unit prices of different genotyping methods

Method	Cost ($)
SNaPshot	2
PCR-RFLP	0.52
Visual detection method	0.5

## 2 Materials and methods

### 2.1 Animals

The samples used in this study were collected at a slaughterhouse in Shihezi, Xinjiang, China. Two samples were collected from the uterus of two pregnant ewes. There was one lamb in the uterus of one ewe and three lambs in the uterus of the other ewe. About 3 g of uterus samples of the two different ewes were dissected and stored. In addition, samples of 47 hybrid Kazakh sheep (offspring produced by mating purebred Kazakh sheep with other potentially high-yielding sheep) were randomly collected, and about 3 g of muscle tissue samples were dissected from them. All samples were labeled and stored in cryopreserved tubes in liquid nitrogen.


**Ethical approval:** The research related to animal use has been complied with all the relevant national regulations and institutional policies for the care and use of animals and has been approved by the Animal Protection and Use Committee of Shihezi University (SUACUC-08032). All procedures performed in studies involving animals were in accordance with the ethical standards of the “Regulations for the Management of Laboratory Animals” (Chinese State Council No. 676, revised in March 2017).

### 2.2 DNA extraction

The genomic DNA of the sample was extracted using a Genomic DNA extraction kit (Tiangen Biochemical Technology Co., Ltd, Beijing, China). With the exception of the DNA on the adsorption column, which was washed with water, other extraction steps were carried out according to the manufacturer’s instructions. The extracted DNA was examined by agarose gel electrophoresis, and a spectrophotometer (ND2000; Thermo Fisher Scientific, USA) was used to determine its purity and quality. The extracted DNA samples were stored at −20°C.

### 2.3 ARMS

**Figure 1:**
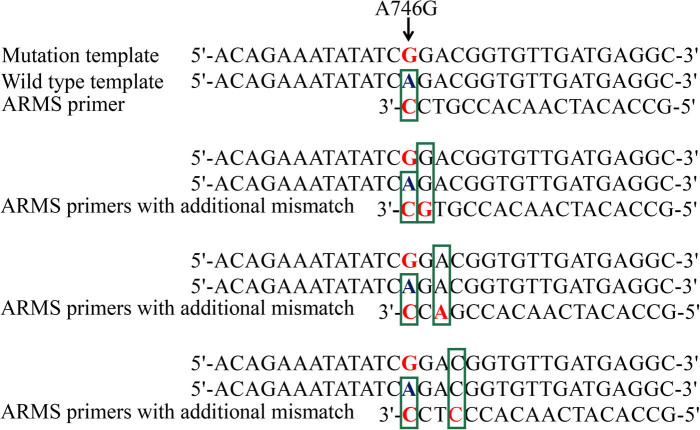
Design principle of ARMS primers for the *FecB* gene.

Using the gene sequence of *FecB* (NC_040257.1) published by GenBank, Prime premier 5.0 (http://www.downzai.com/soft/7003.html) was used to design ARMS specific primers for the *FecB* gene, which were then used to amplify the sequence of the mutation genotype (rs418841713, A > G) without amplifying the sequence of the wild type (Figure 1). Sometimes, a mismatch of a single base does not affect the progress of the PCR, but the PCR is hindered by the difficulty in phosphodiester bond formation when there are two juxtaposed mismatches. Therefore, we artificially designed a mismatch at bases 2–4 of the 3′ end of the reverse primer to screen for the most specific primer pair (Figure 1 and Table 3). The forward primers are the same for all primer pairs (Table 3). The ARMS specific primer pair would only amplify the BB or B+ genotype, rather than the ++ genotype.

**Table 3:** Sequences of ARMS primers used in this study

Primer name		Sequence (5′–3′)	Mismatched position (distance from the 3′ end)	Annealing temperature
FI	Forward primer	AACTTGTCTCACCAGTCTCCT	—	62°C
	Reverse primer	GCCTCATCAACACCGTCC		
FCII	Forward primer	AACTTGTCTCACCAGTCTCCT	2	64°C
	Reverse primer	GCCTCATCAACACCGTGC		
FCⅢ	Forward primer	AACTTGTCTCACCAGTCTCCT	3	62°C
	Reverse primer	GCCTCATCAACACCGACC		
FCIV	Forward primer	AACTTGTCTCACCAGTCTCCT	4	64°C
	Reverse primer	GCCTCATCAACACCCTCC		

Note: Due to the characteristics of ARMS-specific primers, the forward primers for all primer pairs are identical.

In addition, we also designed the classic primers for the A746G SNP site, that is, the primers containing the SNP site in the middle instead of the 3′ end of the reverse primer. The primers were designed as follows: forward primer: 5′-TCCAGAGGACGATAGCAAAGC-3′, reverse primer: 5′-GTGAGGTCTCCCATTAGAAGCA-3′, and the product size was 365 bp. And then the classic primers were used to amplify the samples, and the PCR products were sent to Beijing Ruibo Xingke Biotechnology Co., Ltd (Beijing, China) for sequencing to verify the genotype of the samples. As the *FecB* has the A → G mutation at position 746, if the sequencing result at this site was A, we assumed it was the ++ genotype, and if the sequencing result at this site was G, we assumed it was the BB genotype, and if there were two peaks in the sequencing result, we assumed it was the B+ genotype.

Samples of two ewes (known for their litter size) were amplified using the above-mentioned classic PCR primers, and the PCR products were sequenced to obtain their genotypes. Each reaction mixture contained the following: 10 µL of 2× Taq PCR Mix (Tiangen Biochemical Technology Co., Ltd, Beijing, China), 0.5 µM each of the forward and reverse primer, 1 µL of the DNA template, and finally sterile water was added to increase the reaction volume to 20 µL. The PCR conditions were as follows: 5 min at 95°C, 30 cycles of 30 s at 95°C, 30 s at 61°C and 30 s at 72°C; 5 min at 72°C, and the amplified product was stored at 4°C.

If the two samples had the BB or B+ genotype and the ++ genotype, then the two samples were amplified by PCR with the designed ARMS specific primers. After testing the ARMS PCR amplification system, each ARMS reaction mixture contained the following: 0.1 µL of Ex Taq (5 U/µL; TaKaRa Biotechnology Co., Ltd, Beijing, China), 2 µL of 10× PCR buffer (Mg^2+^ free), 1.2 µL of MgCl_2_ (25 mM), 1.6 µL of dNTP mixture (2.5 mM each), 0.5 µM of each forward and reverse primer, 1 µL of DNA template, and finally sterile water was added to increase the reaction volume to 20 µL. The ARMS PCR conditions were as follows: 4 min at 95°C, 30 cycles of 10 s at 98°C, 10 s at different annealing temperatures shown in Table 3 and 20 s at 72°C; 5 min at 72°C. This program enabled us to complete the ARMS PCR in about 50 min, and the PCR can be used in the field with the help of a hand-held PCR thermocycler.

### 2.4 ARMS visual detection

Visual detection of *FecB* expression can provide a better basis for the breeding of BB or B+ genotype sheep. At present, the method for detecting ARMS results is agarose gel electrophoresis. In comparison to other methods, the nucleic acid dye staining method can not only improve the resolution of detection by the naked eye, but is also expected to be used directly for on-site detection. It is the most simple and convenient way to determine the result. After ARMS amplification, 8.57 µL of 100× SYBR Green I (1:100 diluted 10,000× concentrated solution; Solarbio Science & Technology Co., Ltd, Beijing, China) was added directly to the 20 µL of ARMS product so that the final dye concentration was 30×, which can then be observed by the naked eye. For BB or B+ genotype samples (the genomic DNA concentration was 102 ng/µL and 90 ng/µL, respectively), because of the amplification of ARMS specific primer pairs and templates, the amplification products were combined with SYBR Green I and the amplification products showed bright green fluorescence. In contrast, the ++ genotype sample could not be combined with ARMS specific primers to produce PCR reaction, so the reaction system showed the original color of SYBR Green I, which is orange yellow. We used samples of known BB genotype and ++ genotype ewes for ARMS visual detection, and ddH_2_O replaced the template as a negative control. These results were identified by the naked eye and photographed and recorded using a smartphone.

### 2.5 Accuracy and sensitivity of ARMS visual detection

To test the accuracy of this method, DNA of 47 Kazakh sheep samples was extracted using a genomic DNA extraction kit. ARMS PCR was performed with 49 samples (including samples of known BB genotype and ++ genotype ewes as control, which were used in the previous section for ARMS). Then, SYBR Green I was added to the PCR products of each sample, and the color of the PCR products in the PCR tube was observed immediately after mixing. The results were recorded using a smartphone. Then, 2.5 µL of 10× loading buffer (TaKaRa Biotechnology Co., Ltd, Beijing, China) was added to the PCR tube, and agarose gel electrophoresis was performed to preliminarily verify the results of naked eye judgment. In total, 47 samples were then sequenced using classic primers to verify the accuracy of the ARMS visual inspection.

In order to examine the sensitivity of this method, the DNA template from the known BB genotype was selected and diluted with 10 times gradient, that is, the diluted template gradient was 5, 0.5, 0.05, 0.005 and 0.0005 ng/µL. Detection by the naked eye was then performed, and the PCR products were verified by agarose gel electrophoresis.

## 3 Results

### 3.1 Screening of ARMS optimal primers

First, in order to evaluate the specificity of the ARMS primers, an ARMS specific primer pair was designed for the SNP loci of the *FecB*, and the 3′ end of the reverse primer was complementary to the SNP loci of the *FecB*. In addition to this, a mismatch was designed at positions 2, 3 and 4 from the 3′ end of the reverse primer to enhance the specificity of the primer pairs. The specific sequences of the primers are shown in Table 3; their forward primers are all the same, and these four primer pairs were used for subsequent research.

**Figure 2:**
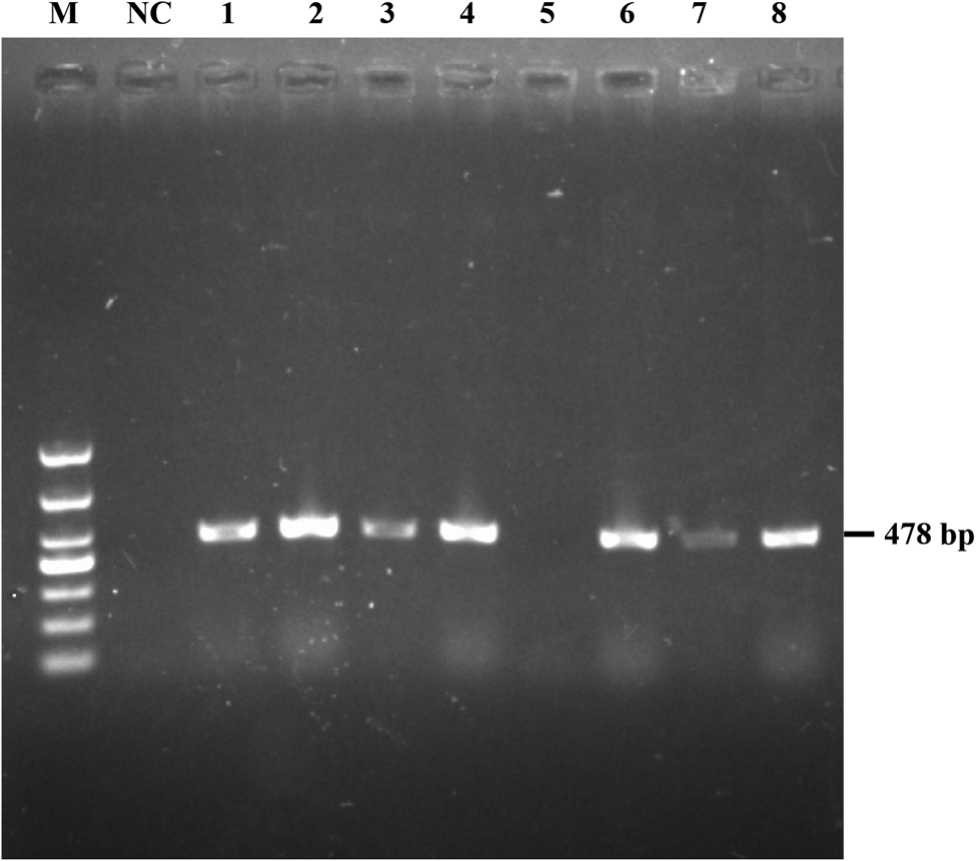
Screening of ARMS optimal primer pairs. M: DL1000 DNA marker; NC: negative control; PCR product size is 478 bp; 1 and 2: the lanes for the amplification products of the template of ++ and BB genotypes, respectively, with primer FI (without additional mismatch); 3 and 4: the lanes for the amplification products of the template of ++ and BB genotypes, respectively, with primer FII (with an additional mismatch in the second position at the 3′ end of the reverse primer); 5 and 6: the lanes for the amplification products of the template of ++ and BB genotypes, respectively, with primer FCIII (with an additional mismatch in the third position at the 3′ end of the reverse primer); 7 and 8: the lanes for the amplification products of the template of ++ and BB genotypes, respectively, with primer FCIV (with an additional mismatch in the fourth position at the 3′ end of the reverse primer).

After the ARMS PCR amplification of the ++ and BB genotype templates, 5 µL of ARMS product from each sample was subjected to agarose gel electrophoresis to select the primers with the best reaction. The results (Figure 2) showed that all primer pairs could be amplified for the BB genotype. The PCR product size was 478 bp, which was in line with the expected length. However, when there was an additional mismatch at the second (FCII) or fourth (FCIV) base from the 3′ end of the reverse primer, the primer pairs could still amplify a very shallow band for the ++ genotype. When there was no additional mismatch of the reverse primer (FCI), a more obvious band was amplified for the ++ genotype. Strikingly, the primer pair (FCIII) with an additional mismatch at the third base at the 3′ end of the reverse primer generated a higher level of amplification. This pair of primers amplified the BB genotype, but not the ++ genotype. Therefore, the primer pair FCIII was used for subsequent experiments, including visual detection and its accuracy and sensitivity inspection.

### 3.2 ARMS visual detection

**Figure 3:**
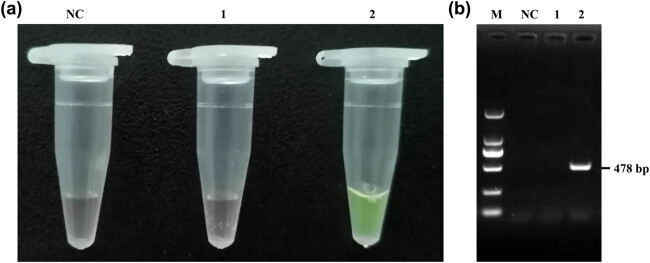
ARMS visual detection of *FecB.* (a) Visual detection of the sample of known ++ and BB genotypes. (b) Electrophoresis verification results of visual detection. M: DL2000 DNA marker; NC: negative control; (1) ++ genotype; (2) BB genotype.

The detection of ARMS amplification was performed by naked eye examination with the support of gel electrophoresis analysis. The primer pair FCIII was selected for ARMS PCR amplification with DNA templates of known ++ and BB genotype sheep. Thus, the ++ genotype template and no template was used as positive and negative controls, respectively. 8.57 µL of 100× SYBR Green I was added to the 20 µL of ARMS PCR product to give a final dye concentration of 30× after the ARMS reaction. The fluorescence results were observed immediately with the naked eye. The ++ genotype sheep sample appeared orange yellow, while the BB genotype sample appeared bright green, as expected (Figure 3). Clear differences were observed between the ++ and the BB genotype sheep samples, demonstrating the robustness of the magnified visual detection of ARMS. Moreover, the fluorescence in the PCR product lasted for an appreciable time; as long as the PCR product does not degrade, the fluorescence will always be visible.

### 3.3 Accuracy of ARMS visual detection

**Figure 4:**
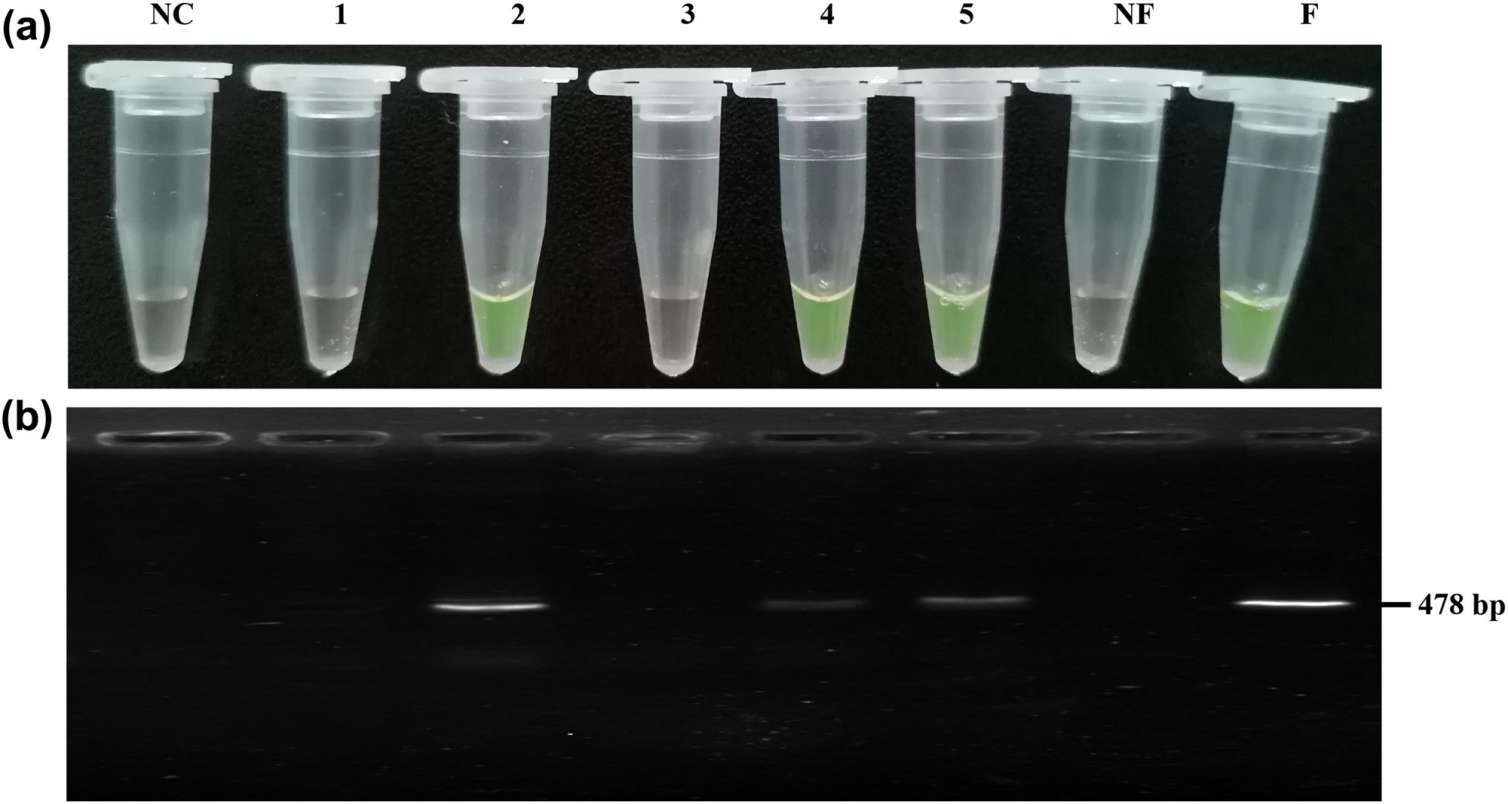
ARMS visual detection results of five samples from 47 hybrid Kazakh sheep. (a) Visual detection of the unknown five samples. (b) Electrophoresis verification results of visual detection. NC: negative control; 1–5 are the samples to be detected, 1 and 3 were detected as the ++ genotype, whereas 2, 4 and 5 were detected as the BB genotype; NF: the known ++ genotype; F: the known BB genotype.

In order to explore the feasibility of future application of this method, such as instant detection, 47 hybrid Kazakh sheep samples were collected for ARMS amplification and visual detection (Table S1) to verify the accuracy of the method. In detail, after performing ARMS specific amplification of the DNA of 47 sheep samples, 8.57 µL of 100× SYBR Green I was directly added to the tube containing the amplified product. The ++ and BB genotype sheep templates were used as references, and no template was added as the negative control. The fluorescence results were observed immediately with the naked eye (Figure 4 and Figure S1). The results suggested significant color variations between the BB or the B+ genotype and the ++ genotype, which could be easily seen with the naked eye. This further demonstrates the reproducibility and stability of ARMS visual detection.

**Table 4:** The accuracy of ARMS visual detection

Visual detection	Sequencing analysis		Total	Accuracy
	++ genotype	BB or B+ genotype		
++ genotype	26	0	26	100%
BB or B+ genotype	0	21	21	
Total	26	21	47	

At the same time, the 47 sheep samples were subjected to sequencing and used as a gold standard to verify the accuracy of the ARMS visual detection. The sequencing results were completely consistent with the results obtained by ARMS visual detection (Table 4, Table S1 and Figure S2), which means that the method is considered 100% accurate. This kind of instant visual detection method shows considerable promise for direct on-site detection. For example, we can use a hand-held PCR instrument to perform on-site detection of the BB or the B+ genotype for providing a reference for sheep breeding.

### 3.4 Sensitivity of ARMS visual detection

**Figure 5:**
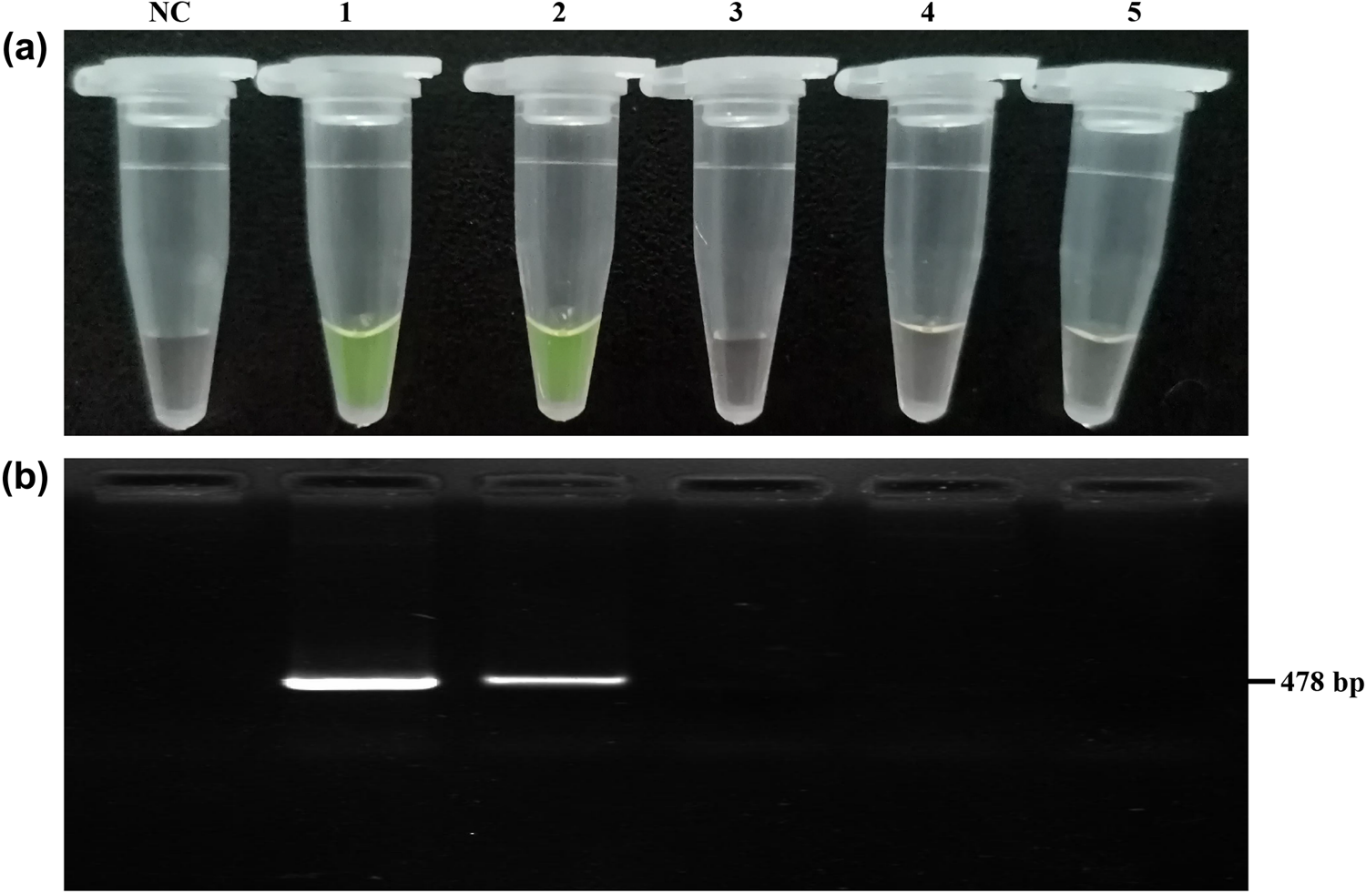
ARMS visual detection sensitivity examination. (a) Visual detection of the known BB genotype. (b) Electrophoresis verification results of visual detection. NC: negative control; 1–5 represent the template concentration of the known BB genotype after 10 times gradient dilution: 5, 0.5, 0.05, 0.005 and 0.0005 ng/µL.

Next, the sensitivity of the ARMS visual detection method was examined. ARMS visual detection was performed after diluting the DNA of the known BB genotype. The results showed that no green fluorescence could be observed in the PCR tube when the concentration of the template was lower than 0.5 ng/µL (Figure 5a). It was further confirmed by agarose gel electrophoresis that no band was observed when the concentration of the template was below 0.5 ng/µL (Figure 5b). Therefore, the ARMS visual detection has a sensitivity of 0.5 ng/µL.

## 4 Discussion

Compared with conventional methods for detecting the *FecB* gene in sheep, ARMS detection provided better reproducibility and more accurate results. The method has been successfully applied to SNP analysis [21], and it also can be used to detect the *FecB* gene with a single base mutation. However, in some cases, although the annealing temperature is relatively high, the PCR can still proceed smoothly in the presence of a single base mismatch, so it cannot be applied to the detection of SNP. To solve this problem, we used an improved ARMS method that introduced additional base pair changes in the last four bases of the 3′ end of the reverse primer [26,27,28]. The introduction of additional mismatches made it difficult to amplify non-specific alleles, but it had little effect on the amplification of specific alleles [29,30]. The additional mismatches markedly increased the specificity of primers [31].

It has been proposed [21] that an additional mismatch at the fourth or third base of the 3′ end of the reverse primer can be well used for identification. In this study, we attempted to introduce an additional mismatch at the second to fourth bases of the 3′ end of the reverse primer. The results showed that only one mismatched primer pair not only can amplify the BB or the B+ genotype DNA templates, but also can amplify the ++ genotype DNA templates with low efficiency. However, for the primer pair with an additional mismatch, the primers contain two mismatches for the wild type template, but only one mismatch for the BB or the B+ genotype templates. Therefore, they only amplified the BB or the B+ genotype templates, but not the wild type templates. In particular, the specificity was significantly increased when there was an additional mismatch at the third base of the 3′ end of the reverse primer.

At present, the classical method for detecting ARMS PCR is agarose gel electrophoresis, which is quite complicated and also requires equipment such as electrophoresis apparatus. In comparison, the dye analysis method not only improves the resolution of detection by the naked eye, but also is expected to be applied to field detection, which is the simplest and the most convenient way to examine the result. This study chose to use SYBR Green I as the nucleic acid dye. After adding the dye to the tube containing the PCR product, we can immediately examine whether there is an amplification product by observing the color change in the PCR tube and examine whether the sample is a mutation genotype (BB or B+) or a wild type (++) genotype. SYBR Green I was used to detect ARMS amplification [25]. It binds to the minor groove region of double stranded DNA (dsDNA). The dye emits very weak fluorescence in the free state; however, when it is combined with the dsDNA, the fluorescence intensity is greatly enhanced. Therefore, the change in fluorescence intensity can be utilized to detect nucleic acid amplification. In addition, when the concentration of SYBR Green I is high, it is orange yellow under natural light conditions and bright green when it binds to dsDNA. If the test sample carries the *FecB* gene (BB or B+), the resulting dsDNA product will bind to SYBR Green I after amplification with ARMS-specific primers, and the dye color will turn bright green. As the amount of product increases, the green fluorescence becomes more pronounced. However, because the ++ genotype does not amplify with ARMS-specific primers and does not generate products that can bind to the dye, the reaction system still appears orange yellow. In other words, SYBR Green I was directly added to the ARMS PCR system and whether the sheep carried the *FecB* gene was judged by direct observation of the color change in the reaction system, that is, the BB and the B+ genotypes were bright green, but the ++ genotype was orange yellow. Overall, ARMS visual detection is a more convenient method, which breaks away from the traditional method of detecting ARMS and simplifies the detection steps.

In order to provide a better reference for sheep breeding, it is a basic necessity to make ARMS-based visual detection suitable for field testing. The established visual detection method avoids the use of large instruments for the detection of results. Although, the inevitable thermal cycling equipment makes its application in the field challenging [20]. But it is possible to use a hand-held PCR thermocycler to make the method more suitable for on-site detection [32].

## 5 Conclusions

In this study, the combination of ARMS-PCR and the nucleic acid dye SYBR Green I was successfully used for the robust and convenient visual detection of the sheep *FecB* gene, which is a potential reference for the breeding of multiple births in sheep.

## References

[1] Wang W, La Y, Zhou X, Zhang X, Li F, Liu B. The genetic polymorphisms of TGFβ superfamily genes are associated with litter size in a Chinese indigenous sheep breed (Hu sheep). Anim Reprod Sci. 2018;189:19–29. 10.1016/j.anireprosci.2017.12.003 29274749

[2] Fogarty NM. A review of the effects of the Booroola gene (FecB) on sheep production. Small Ruminant Res. 2009;85(2–3):75–84.

[3] Sudhakar A, Rajendran R, Rahumathulla PS. Detection of Booroola (FecB) mutation in Indian sheep – Nilagiri. Small Ruminant Res. 2013;113(1):55–7.

[4] Piper LR, Bindon BM. The Booroola Merino and the performance of medium non-Peppin crosses at Armidale. Wool Technol Sheep Breed. 1983;31(1):14–9.

[5] Gootwine E, Reicher S, Rozov A. Prolificacy and lamb survival at birth in Awassi and Assaf sheep carrying the FecB (Booroola) mutation. Anim Reprod Sci. 2008;108(3–4):402–11. 10.1016/j.anireprosci.2007.09.009 17997056

[6] Davis GH, Galloway SM, Ross IK, Gregan SM, Ward J, Nimbkar BV, et al. DNA tests in prolific sheep from eight countries provide new evidence on origin of the Booroola (FecB) mutation. Biol Reprod. 2002;66(6):1869–74. 10.1095/biolreprod66.6.1869 12021074

[7] Kumar S, Kolte AP, Mishra AK, Arora AL, Singh VK. Identification of the FecB mutation in Garole × Malpura sheep and its effect on litter size. Small Ruminant Res. 2006;64(3):305–10.

[8] Mishra AK, Arora AL, Kumar S, Prince LLL. Studies on effect of Booroola (FecB) genotype on lifetime ewes’ productivity efficiency, litter size and number of weaned lambs in Garole × Malpura sheep. Anim Reprod Sci. 2009;113(1–4):293–8. 10.1016/j.anireprosci.2008.06.002 18620822

[9] Guan F, Liu SR, Shi GQ, Yang LG. Polymorphism of FecB gene in nine sheep breeds or strains and its effects on litter size, lamb growth and development. Anim Reprod Sci. 2007;99(1–2):44–52. 10.1016/j.anireprosci.2006.04.048 16859845

[10] Asadpour RJ, Joozani RA. Detection of polymorphism in booroola gene (FecB) and its association with litter size in Zel sheep breed in Iran. Slovak J Anim Sci. 2012;45(2):63–6.

[11] Davis GH, Balakrishnan L, Ross IK, Wilson T, Galloway SM, Lumsden BM, et al. Investigation of the Booroola (FecB) and Inverdale (FecXI) mutations in 21 prolific breeds and strains of sheep sampled in 13 countries. Anim Reprod Sci. 2006;92(1–2):87–96. 10.1016/j.anireprosci.2005.06.001 15982834

[12] Chen Y, Luo QJ, Li DZ, Zhang YJ, Yang FY, Yang JQ, et al. Relationship between BMPR-IB polymorphism and litter size in six breeds or strains of sheep. J Xinjiang Agric Univ. 2008;2:12–6.

[13] Kumar S, Mishra AK, Kolte AP, Dash SK, Karim SA, et al. Screening for Booroola (FecB) and Galway (FecXG) mutations in Indian sheep. Small Ruminant Res. 2008;80(1–3):57–61.

[14] Mahdavi M, Nanekarani S, Hosseini SD. Mutation in BMPR-IB gene is associated with litter size in Iranian Kalehkoohi sheep. Anim Reprod. 2014;147(3–4):93–8. 10.1016/j.anireprosci.2014.04.003 24793585

[15] Haile A, Getachew T, Mirkena T, Duguma G, Gizaw S, Wurzinger M, et al. Community-based sheep breeding programs generated substantial genetic gains and socioeconomic benefits. Animal. 2020;14(7):1362–70. 10.1017/S1751731120000269 PMC7301245 32100664

[16] Mulsant P, Lecerf F, Fabre S, Schibler L, Monget P, Lanneluc I, et al. Mutation in bone morphogenetic protein receptor-IB is associated with increased ovulation rate in Booroola Merino ewes. Proc Natl Acad Sci U S A. 2001;98(9):5104–9. 10.1073/pnas.091577598 PMC33171 11320249

[17] Hua GH, Chen SL, Ai JT, Yang LG. None of polymorphism of ovine fecundity major genes FecB and FecX was tested in goat. Anim Reprod Sci. 2008;108(3–4):279–86. 10.1016/j.anireprosci.2007.08.013 17964743

[18] Sun L, Liu Q, Li S, Ma G, Wang Z, Ma C, et al. A new strategy to confirm the identity of tumour tissues using single-nucleotide polymorphisms and next-generation sequencing. Int J Legal Med. 2020;134(2):399–409. 10.1007/s00414-019-02216-9 31811377

[19] Zhang Y, Qu S, Zhao J, Yu T, Guo L, Yin S, et al. A novel RFLP-ARMS TaqMan PCR-based method for detecting the BRAF V600E mutation in melanoma. Oncol Lett. 2018;16(2):1615–21. 10.3892/ol.2018.8844 PMC6036452 30008844

[20] Zhu L, Zhang S, Xun Y, Jiang Y, Xia B, Chen X, et al. Comparison of the Amplification Refractory Mutation System, Super Amplification Refractory Mutation System, and Droplet Digital PCR for T790 M Mutation Detection in non-small cell lung cancer after failure of tyrosine kinase inhibitor treatment. Pathol Oncol Res. 2018;24(4):843–51. 10.1007/s12253-017-0286-3 28868565

[21] Qian L, Ding G, Zhou Q, Feng Z, Ding X, Gu S, et al. Molecular authentication of Dendrobium loddigesii Rolfe by amplification refractory mutation system (ARMS). Planta Med. 2008;74(4):470–3. 10.1055/s-2008-1034360 18484545

[22] Wang YZ, Zhu Z, Zhang HY, Zhu MZ, Xu X, Chen CH, et al. Detection of hepatitis B virus A1762T/G1764A mutant by amplification refractory mutation system. Brazilian J Infect Dis. 2014;18(3):261–5. 10.1016/j.bjid.2013.09.005 PMC9427444 24389280

[23] Pati N, Schowinsky V, Kokanovic O, Magnuson V, Ghosh S. A comparison between SNaPshot, pyrosequencing, and biplex invader SNP genotyping methods: accuracy, cost, and throughput. J Biochem Biophys Methods. 2004;60(1):1–12. 10.1016/j.jbbm.2003.11.005 15236905

[24] Marini M, Sasongko TH, Watihayati MS, Atif AB, Hayati F, Gunadi, et al. Allele-specific PCR for a cost-effective & time-efficient diagnostic screening of spinal muscular atrophy. Ind J Med Res. 2012;135(1):31–5. 10.4103/0971-5916.93421 PMC3307181 22382180

[25] Wang R, Zhang F, Wang L, Qian W, Qian C, Wu J, et al. Instant, visual, and instrument-free method for on-site screening of GTS 40-3-2 soybean based on body-heat triggered recombinase polymerase amplification. Anal Chem. 2017;89(8):4413–8. 10.1021/acs.analchem.7b00964 28345860

[26] Newton CR, Graham A, Heptinstall LE, Powell SJ, Summers C, Kalsheker N, et al. Analysis of any point mutation in DNA. The amplification refractory mutation system (ARMS). Nucleic Acids Res. 1989;17(7):2503–16. 10.1093/nar/17.7.2503 PMC317639 2785681

[27] Kwok S, Kellogg DE, McKinney N, Spasic D, Goda L, Levenson C, et al. Effects of primer-template mismatches on the polymerase chain reaction: human immunodeficiency virus type 1 model studies. Nucleic Acids Res. 1990;18(4):999–1005. 10.1093/nar/18.4.999 PMC330356 2179874

[28] Hayashi K, Hashimoto N, Daigen M, Ashikawa I. Development of PCR-based SNP markers for rice blast resistance genes at the Piz locus. Theor Appl Genet. 2004;108(7):1212–20. 10.1007/s00122-003-1553-0 14740086

[29] Drenkard E, Richter BG, Rozen S, Stutius LM, Angell NA, Mindrinos M, et al. A simple procedure for the analysis of single nucleotide polymorphisms facilitates map-based cloning in Arabidopsis. Plant Physiol. 2000;124(4):1483–92. 10.1104/pp.124.4.1483 PMC1539302 11115864

[30] Kwok S, Chang SY, Sninsky JJ. A guide to the design and use of mismatched and degenerate. Genome Res. 1994;3:S39–47. 10.1101/gr.3.4.s39 8173508

[31] Cha RS, Zarbl H, Keohavong P, Thilly WG. Mismatch amplification mutation assay (MAMA): application to the cH-ras gene. Genome Res. 1992;2(1):14–20. 10.1101/gr.2.1.14 1490171

[32] Emanuel PA, Bell R, Dang JL, McClanahan R, David JC, Burgess RJ, et al. Detection of Francisella tularensis within infected mouse tissues by using a hand-held PCR thermocycler. J Clin Microbiol. 2003;41(2):689–93. 10.1128/JCM.41.2.689-693.2003 PMC149716 12574268

